# Effectiveness of Ectoin lozenges on oropharyngeal allergic symptoms

**DOI:** 10.1002/clt2.12095

**Published:** 2022-01-06

**Authors:** Rawan Khachouk, Ursula Pieper‐Fürst, Cengizhan Acikel, Carina Kolot, Andreas Bilstein, Ralph Mösges

**Affiliations:** ^1^ Institute of Medical Statistics and Computational Biology Faculty of Medicine University of Cologne Cologne Germany; ^2^ ClinCompetence Cologne GmbH Cologne Germany; ^3^ bitop AG Dortmund Germany; ^4^ Ursatec GmbH Tholey Germany

**Keywords:** Ectoin, Ectoin lozenges, oropharyngeal allergic symptoms, study according to §23b German Medical Devices Act

To the editor,

Allergic rhinitis (AR) is a common disease resulting in nasal, ocular and potentially oropharyngeal symptoms.^1^ Up to 70% of pollen allergy patients also suffer from the “oral allergy syndrome” (OAS), manifesting in itching and swelling of the oropharynx.^2^ Antihistamines are the first choice of drugs to treat allergic oropharyngeal symptoms, but reservation against use of drugs and side effects may limit patient compliance.^1^ In addition to pharmacotherapies against AR, guidelines recommend allergen‐specific immunotherapy (SIT), comprising subcutaneous (SCIT) or sublingual (SLIT) forms.^3^ However, in more than 50% of patients, SLIT preparations cause side effects, resulting in oropharyngeal itching, swelling, discomfort or irritation.^4^ These side effects often begin upon initiation of SLIT and last for 30–60 min.^5^ Therapy of these side effects includes antihistamines.^6^

Here, Ectoin^®^ containing lozenges (CE‐marked medical device Ectoin^®^ Allergy Lozenges), a non‐pharmacological treatment option for allergic oropharyngeal symptoms, was investigated. Ectoin^®^ is a compatible solute with membrane‐protecting and inflammation‐reducing properties, whose clinical efficacy in AR has already been reported.^7^

The current study investigated therapeutic and preventive effects of Ectoin^®^ lozenges on oropharyngeal allergic symptoms using the initiation of SLIT as a study model.

The multi‐center, prospective, randomized, controlled study was performed according to §23b German Medical Devices Act (MPG), registered with ClinTrials.gov database (NCT03975257) and approved by the responsible ethics committees. Eligibility criteria are described in Table S1.

Treatment (one lozenge) was administered 5 min before (preventive) or 5 min after (therapeutic) the first SLIT dose. No lozenge was administered in the control group. SLIT was carried out in accordance with the guidelines^6^; Fexofenadine was available as on‐demand rescue medication.

Patients' oropharyngeal symptoms were assessed in a patient questionnaire 30 min after SLIT initiation, determining the perception of allergic symptoms (swelling, itching, and irritation) in mouth, on the lips and in the throat/pharynx. Symptom intensity was evaluated as 0 = “no symptoms”, 1 = “mild symptoms, 2 = “moderate symptoms,” or 3 = “severe symptoms.” The sum score of individual allergic symptoms of an organ resulted in the organ‐specific symptom score, and the sum of organ‐specific symptoms in the oropharyngeal symptom score (OPSS; min = 0, max = 27).

Tolerability and safety of treatments were assessed on the occurrence of (serious) adverse events.

Eighty‐nine patients aged 19–75 years with equal distribution into preventive, therapeutic, or control group were enrolled in seven study sites (Figure S1, Table S2). Treatment groups were balanced regarding gender, age, and type(s) of treated allergy (Table S3). No serious adverse event and one mild adverse event occurred in the study (Table S3).

Preventive and therapeutic treatment with Ectoin^®^ lozenges resulted in reduction of oropharyngeal symptoms compared to the control group, reaching significant differences regarding mouth symptoms and OPSS (Figure 1).

**FIGURE 1:**
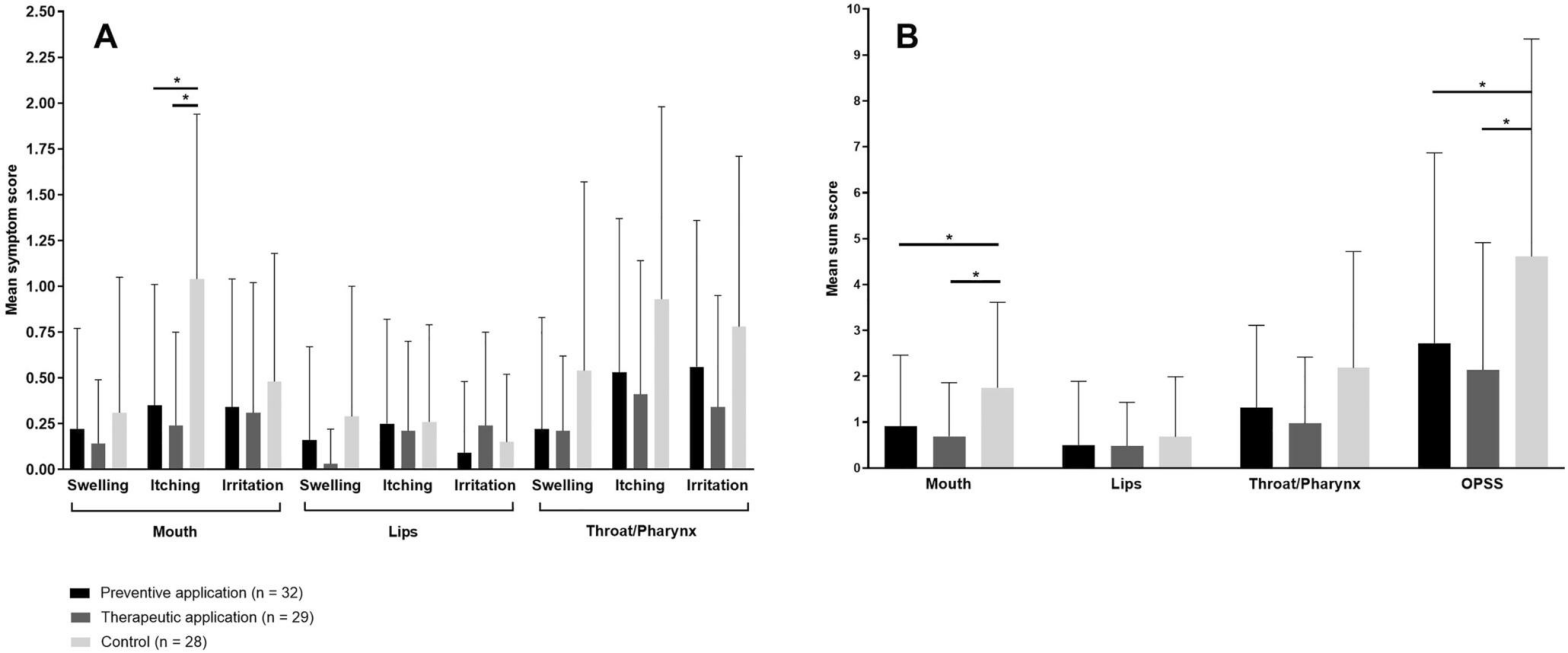
Mean single symptoms (A) and sum symptoms (B) present in different organs following preventive or therapeutic treatment with Ectoin^®^ lozenges compared to a control group. *Statistical significance (*p*‐value < 0.017 after Bonferroni correction). OPSS, oropharyngeal symptom score

The most pronounced symptom in the mouth was itching. Preventive treatment with Ectoin^®^ lozenges resulted in 66% lower itching scores (*p* = 0.002), and patients treated therapeutically showed 77% lower values (*p* = 0.000) compared to control. In line with this, sum scores of the mouth were statistically significantly lower in patients treated with Ectoin^®^ lozenges compared to control (Figure 1).

Swelling and itching of the lips were lower upon preventive and therapeutic treatment compared to control, whereas symptom scores for lip irritation were lower upon preventive treatment but higher upon therapeutic treatment compared to control. Overall, symptoms of the lips were not significantly different between groups (Figure 1).

Pharynx/throat symptoms were lower in patients treated preventively or therapeutically compared to control without significant differences between groups (Figure 1).

Differences in the OPSS were significant (*p* < 0.017) comparing preventive treatment (2.72), therapeutic treatment (2.14) and the control group (4.61; Figure 1).

A subgroup analysis of patients with seasonal allergy demonstrated significantly lower values reflecting itching of the mouth, sum scores of the mouth and OPSS upon preventive and therapeutic treatment (Figure S2). Further, significantly lower values of itching and sum scores of the throat were observed upon therapeutic treatment (Figure S2).

Taken together, Ectoin^®^ lozenges, when applied as preventive or therapeutic treatment upon SLIT, reduced oropharyngeal allergic symptoms without resulting in safety concerns.

SLIT was used as model system, which may be representative for patients suffering from AR or OAS. Of note, applied SLIT preparations were intentionally not revealed as the study did not aim to study the efficacy of the SLIT treatment itself. Therefore, no conclusion on potential variations introduced through different allergens or different SLIT preparations can be drawn. Importantly, the distribution of allergy types within the treatment groups was comparable and treatment was assigned randomly, thereby limiting the chances of unequal distribution.

One drawback of the current study is the lack of a placebo group. As the study was carried out as §23b medical device study according to the MPG, the application of certified medical devices within their intended use was mandatory, thus obviating a placebo group. Although no comparison between Ectoin^®^ containing lozenges and antihistamine treatment was made in the current study, conclusions may be drawn from another study, investigating treatment of AR with Ectoin^®^ nasal spray and eye drops compared to antihistamine containing nasal spray and eye drops.^8^ Results showed that nasal symptoms decreased upon both treatment regimens without differences between groups, thus indicating a comparable efficacy of treatments. To validate this finding, a future study on Ectoin^®^ lozenges with an active comparator should be performed.

## FUNDING INFORMATION

bitop AG

## CONFLICT OF INTERESTS

Rawan Khachouk and Ursula Pieper‐Fürst have nothing to declare. Carina Kolot and AB were employees of bitop AG at the time the study was carried out. Andreas Bilstein reports personal fees from bitop AG. Ralph Mösges reports grants and non‐financial support from bitop AG during the conduct of the study; personal fees from ALK, grants from ASIT biotech, personal fees from allergopharma, personal fees from Allergy Therapeutics, grants and personal fees from Bencard, grants from Leti, grants, personal fees and non‐financial support from Lofarma, non‐financial support from Roxall, grants and personal fees from Stallergenes, grants from Optima, personal fees from Friulchem, personal fees from Hexal, personal fees from Servier, personal fees from Klosterfrau, non‐financial support from Atmos, personal fees from Bayer, non‐financial support from Bionorica, personal fees from FAES, personal fees from GSK, personal fees from MSD, personal fees from Johnson&Johnson, personal fees from Meda, personal fees and non‐financial support from Novartis, non‐financial support from Otonomy, personal fees from Stada, personal fees from UCB, non‐financial support from Ferrero, grants from Hulka, personal fees from Nuvo, grants from Ursapharm, personal fees from Menarini, personal fees from Mundipharma, personal fees from Pohl‐Boskamp, grants from Inmunotek outside the submitted work.

## Supporting information

TABLE S1

TABLE S2

TABLE S3

FIGURE S1

FIGURE S2
