# Supplementary material for: Effectiveness of Ectoin lozenges on oropharyngeal allergic symptoms
Source: Clin Transl Allergy. 2022 Jan 6;12(1):e12095. doi: 10.1002/clt2.12095 (PMC8738077; doi:10.1002/clt2.12095)
Supplement: Supplementary file 1 — TABLE S1 [file CLT2-12-e12095-s003.docx]

Table S1: Eligibility criteria of patients.

| **Inclusion criteria** | **Exclusion criteria** |
| --- | --- |
| Signed informed consent | Condition after surgery or injury to mouth and throat |
| Female and male patients aged ≥18 years | Hypersensibility to any ingredient of the lozenges |
| Medical indication of a SLIT and planned start of SLIT therapy | Pregnant or breastfeeding women |
