# Supplementary material for: Effectiveness of Ectoin lozenges on oropharyngeal allergic symptoms
Source: Clin Transl Allergy. 2022 Jan 6;12(1):e12095. doi: 10.1002/clt2.12095 (PMC8738077; doi:10.1002/clt2.12095)
Supplement: Supplementary file 2 — TABLE S2 [file CLT2-12-e12095-s005.docx]

Table S2: Details of participating investigators.

|  | **Specification of participating investigators** | **Location of study site** |
| --- | --- | --- |
| 1 | Specialist in ear, nose and throat medicine and allergy | Heidelberg  Germany |
| 2 | Specialist in pulmonary and bronchial medicine and allergy | Bonn  Germany |
| 3 | Specialist in ear, nose and throat medicine | Bad Schönborn  Germany |
| 4 | Specialist in ear, nose and throat medicine and allergy | Jülich  Germany |
| 5 | Specialist in ear, nose and throat medicine | Schorndorf  Germany |
| 6 | Specialist in ear, nose and throat medicine | Aachen  Germany |
| 7 | Specialist in ear, nose and throat medicine and allergy | Duisburg  Germany |
