# Supplementary material for: Effectiveness of Ectoin lozenges on oropharyngeal allergic symptoms
Source: Clin Transl Allergy. 2022 Jan 6;12(1):e12095. doi: 10.1002/clt2.12095 (PMC8738077; doi:10.1002/clt2.12095)
Supplement: Supplementary file 3 — TABLE S3 [file CLT2-12-e12095-s004.docx]

Table S3: Demographic and clinical characteristics and adverse events of patients in each treatment group (preventive or therapeutic application of Ectoin^®^ lozenges compared to a control group). n = number of patients; n.a. = not applicable; early blooming trees comprising birch, hazel, and alder.

| **Patients** | | **Preventive treatment** | **Therapeutic treatment** | **Control group** | **Total** |
| --- | --- | --- | --- | --- | --- |
| Patient allocation | n | 32 | 29 | 28 | 89 |
|  | % | 36.0% | 32.6% | 31.5% | 100% |
| **Gender** | | | | | |
| Female | n | 20 | 14 | 16 | 50 |
|  | % | 62.5% | 48.3% | 57.1% | 56.2% |
| Male | n | 12 | 15 | 12 | 39 |
|  | % | 37.5% | 51.7% | 42.9% | 43.8% |
| **Age (mean)** | | | | | |
|  | years | 39.96 | 35.86 | 38.75 | 38.24 |
| **Diagnosis of type 1 allergy with SLIT indication** | | | | | |
| Early blooming trees | n | 12 | 11 | 10 | 33 |
|  | % | 37.5% | 37.9% | 35.7% | 37.1% |
| Grass pollen | n | 7 | 8 | 5 | 20 |
|  | % | 21.9% | 27.6% | 17.9% | 22.5% |
| House dust mites | n | 13 | 10 | 13 | 36 |
|  | % | 40.6% | 34.5% | 46.4% | 40.4% |
| **Allergy type** | | | | | |
| Seasonal allergy | n | 13 | 14 | 10 | 37 |
|  | % | 40.6% | 48.3% | 35.7% | 41.6% |
| Perennial allergy | n | 4 | 4 | 2 | 10 |
|  | % | 12.5% | 13.8% | 7.1% | 11.2% |
| Mixed allergy | n | 15 | 11 | 16 | 42 |
|  | % | 46.9% | 37.9% | 57.1% | 47.2% |
| **Asthmatic patients** | | | | | |
|  | n | 2 | 2 | 4 | 8 |
|  | % | 6.3% | 6.6% | 14.3% | 9% |
| **Adverse events** | | | | | |
|  | n | 0 | 1 | 0 | 1 |
|  | % | 0.0% | 3.4% | 0.0% | 1.1% |
| Details |  | n.a. | stomatal discomfort after intake of Ectoin^®^ lozenges | n.a. |  |
| Intensity, relationship to treatment |  |  | Mild, possibly related |  |  |
| Outcome |  |  | Ended on the day of the treatment |  |  |
