# Supplementary figures and images for: Effectiveness of Ectoin lozenges on oropharyngeal allergic symptoms
Source: Clin Transl Allergy. 2022 Jan 6;12(1):e12095. doi: 10.1002/clt2.12095 (PMC8738077; doi:10.1002/clt2.12095)

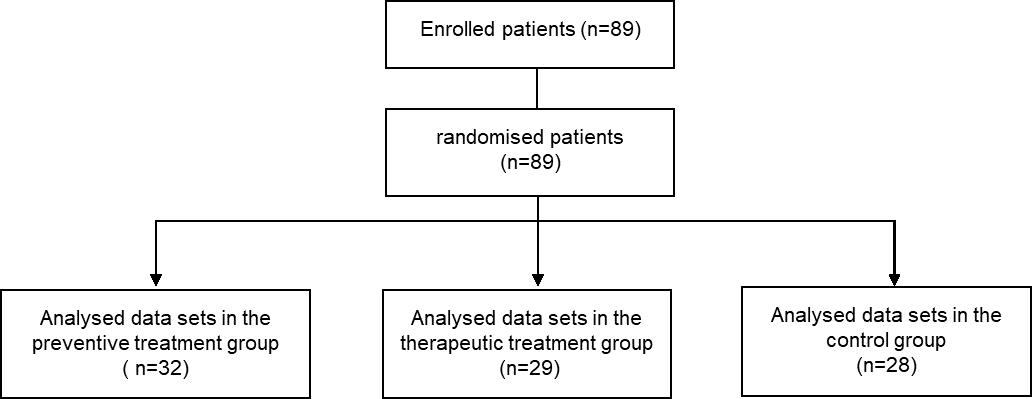

Supplement: Supplementary file 4 — FIGURE S1 [file CLT2-12-e12095-s001.png]

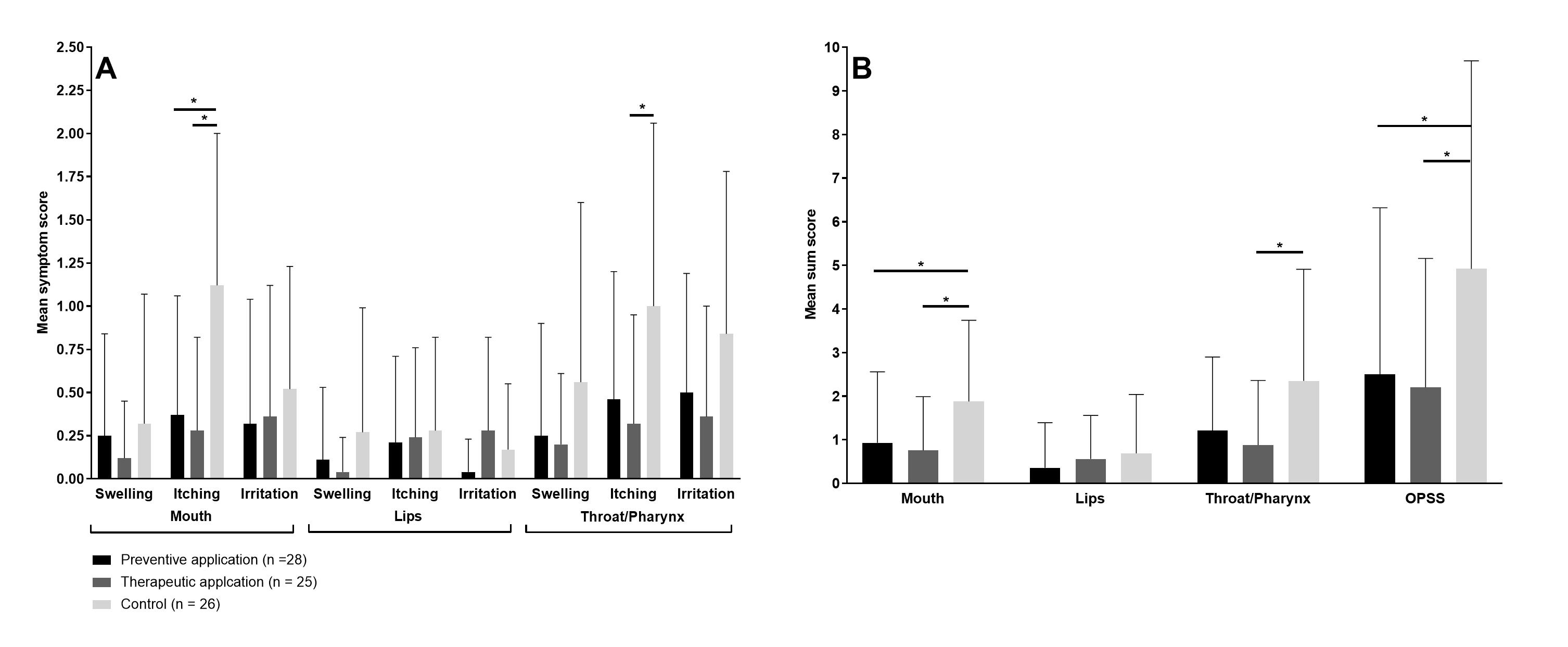

Supplement: Supplementary file 5 — FIGURE S2 [file CLT2-12-e12095-s002.jpg]
